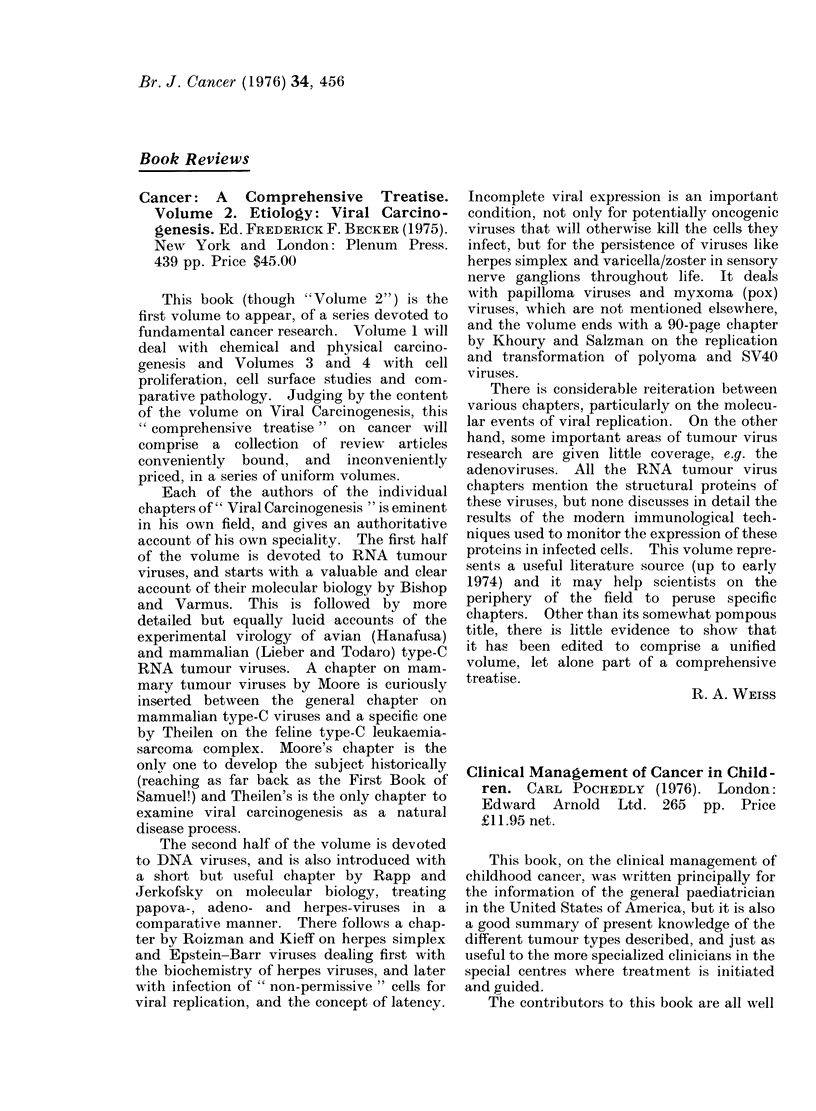# Cancer: A Comprehensive Treatise. Volume 2. Etiology: Viral Carcinogenesis

**Published:** 1976-10

**Authors:** R. A. Weiss


*Br. J. Cancer* (1976) **34**, 456

### *Book Reviews*

**Cancer: A Comprehensive Treatise. Volume 2. Etiology: Viral Carcinogenesis.** Ed. FREDERICK F. BECKER (1975). New York and London: Plenum Press. 439 pp. Price $45.00

This book (though "Volume 2") is the first volume to appear, of a series devoted to fundamental cancer research. Volume 1 will deal with chemical and physical carcinogenesis and Volumes 3 and 4 with cell proliferation, cell surface studies and comparative pathology. Judging by the content of the volume on Viral Carcinogenesis, this " comprehensive treatise " on cancer will comprise a collection of review articles conveniently bound, and inconveniently priced, in a series of uniform volumes.

Each of the authors of the individual chapters of " Viral Carcinogenesis " is eminent in his own field, and gives an authoritative account of his own speciality. The first half of the volume is devoted to RNA tumour viruses, and starts with a valuable and clear account of their molecular biology by Bishop and Varmus. This is followed by more detailed but equally lucid accounts of the experimental virology of avian (Hanafusa) and mammalian (Lieber and Todaro) type-C RNA tumour viruses. A chapter on mammary tumour viruses by Moore is curiously inserted between the general chapter on mammalian type-C viruses and a specific one by Theilen on the feline type-C leukaemia-sarcoma complex. Moore's chapter is the only one to develop the subject historically (reaching as far back as the First Book of Samuel!) and Theilen's is the only chapter to examine viral carcinogenesis as a natural disease process.

The second half of the volume is devoted to DNA viruses, and is also introduced with a short but useful chapter by Rapp and Jerkofsky on molecular biology, treating papova-, adeno- and herpes-viruses in a comparative manner. There follows a chapter by Roizman and Kieff on herpes simplex and Epstein–Barr viruses dealing first with the biochemistry of herpes viruses, and later with infection of " non-permissive " cells for viral replication, and the concept of latency. Incomplete viral expression is an important condition, not only for potentially oncogenic viruses that will otherwise kill the cells they infect, but for the persistence of viruses like herpes simplex and varicella/zoster in sensory nerve ganglions throughout life. It deals with papilloma viruses and myxoma (pox) viruses, which are not mentioned elsewhere, and the volume ends with a 90-page chapter by Khoury and Salzman on the replication and transformation of polyoma and SV40 viruses.

There is considerable reiteration between various chapters, particularly on the molecular events of viral replication. On the other hand, some important areas of tumour virus research are given little coverage, *e.g.* the adenoviruses. All the RNA tumour virus chapters mention the structural proteins of these viruses, but none discusses in detail the results of the modern immunological techniques used to monitor the expression of these proteins in infected cells. This volume represents a useful literature source (up to early 1974) and it may help scientists on the periphery of the field to peruse specific chapters. Other than its somewhat pompous title, there is little evidence to show that it has been edited to comprise a unified volume, let alone part of a comprehensive treatise.

R. A. WEISS